# Effect of a School-Based Physical Activity and Multi-Micronutrient Supplementation Intervention on Cognitive Function and Academic Achievement Among Schoolchildren in Tanzania: Secondary Outcome from the KaziAfya Cluster-Randomized Controlled Trial

**DOI:** 10.3390/ijerph22091335

**Published:** 2025-08-27

**Authors:** Elihaika G. Minja, Emmanuel C. Mrimi, Winfrida P. Mponzi, Johanna Beckmann, Marceline F. Finda, Fredros O. Okumu, Kurt Z. Long, Christin Lang, Jürg Utzinger, Markus Gerber

**Affiliations:** 1 Environmental Health and Ecological Sciences Department, Ifakara Health Institute, Ifakara, P.O. Box 53, Tanzania; emrimi@ihi.or.tz (E.C.M.); wmponzi@ihi.or.tz (W.P.M.); lfinda@ihi.or.tz (M.F.F.); fredros@ihi.or.tz (F.O.O.); 2 Swiss Tropical and Public Health Institute, Kreuzstrasse 2, CH-4123 Allschwil, Switzerland; kurt.long@swisstph.ch (K.Z.L.); juerg.utzinger@swisstph.ch (J.U.); 3 Faculty of Medicine, University of Basel, Petersplatz 1, CH-4003 Basel, Switzerland; 4 Department of Sport, Exercise and Health, University of Basel, Grosse Allee 6, CH-4052 Basel, Switzerland; johanna.beckmann@unibas.ch (J.B.); christin.lang@unibas.ch (C.L.); markus.gerber@unibas.ch (M.G.); 5 School of Life Science and Bioengineering, Nelson Mandela African Institution of Science & Technology, Arusha, P.O. Box 447, Tanzania; 6 School of Biodiversity, One Health and Veterinary Medicine, University of Glasgow, Glasgow G12 8QQ, UK

**Keywords:** academic performance, cluster-randomized trial, cognitive function, micronutrients supplementation, physical activity, school-age children, Tanzania

## Abstract

Background: Physical activity (PA) and adequate micronutrient intake are essential for brain development and may influence cognitive function and academic achievement. However, few large-scale studies have assessed the combined effects of PA and multi-micronutrient supplementation (MMNS) in school-age children. Methods: A cluster-randomized placebo-controlled trial in four peri-urban Tanzanian schools assigned children to one of four groups: (i) PA alone; (ii) MMNS alone; (iii) PA plus MMNS; or (iv) placebo. Children were followed over two school years with assessments at baseline, 14 months, and 26 months. Cognitive function was assessed using computerized Flanker tasks. Academic achievement was evaluated through end-of-year exams in mathematics and Kiswahili subject scores. Anthropometric measures determined nutritional status. Data were analyzed using generalized estimated equations (GEE). Results: Complete data from 559 children (326 girls, 233 boys) aged 6–12 years showed differing characteristics across groups, particularly age and body mass index. No significant intervention effects on cognitive function were found. MMNS groups (alone or combined with PA) showed significantly higher Kiswahili scores, while PA alone had the lowest performance compared to placebo. No intervention effect was found in mathematics. Sex, hemoglobin level, and baseline measures were key predictors for cognition or academic performance. Conclusions: PA and MMNS interventions showed no significant cognitive or academic improvements versus placebo. Further research should optimize school-based nutrition and PA programs for improved learning outcomes.

## 1. Introduction

Cognitive function encompasses a range of abilities, including reasoning, problem-solving, planning, abstract thinking, comprehension of complex ideas, and learning from experience, all of which reflect general intellectual capacity [1]. Middle childhood (ages 6–12 years) represents a particularly important period for both cognitive and social development. During this stage, children acquire foundational academic skills such as reading and mathematics, while also learning essential competencies through interactions with peers and adults [1]. Furthermore, this developmental window allows for the integration of broader cultural and societal values as children continuously engage with their social and physical environments, thereby promoting both cognitive and social growth [2].

While early cognitive development during the preschool years is a strong predictor of later academic success, middle childhood remains a critical period for supporting cognitive development due to continued brain maturation and plasticity. The cognitive function of school-age children is shaped by multiple interrelated factors, including their nutritional status [3], physical activity (PA) [4], and demographic and socioeconomic conditions [5].

Globally, more than 149 million children under the age of 5 years are stunted in terms of linear growth, placing them at risk for delayed physical and mental development [6]. Low- and middle-income countries (LMICs; defined by the World Bank based on gross national income per capita), such as Tanzania, face widespread issues of nutritional deficiencies and frequent infections among school-age children [7,8,9]. These conditions significantly hinder cognitive, motor, and behavioral development. Contributing factors include poor nutrition, infectious diseases, low socioeconomic status (SES), limited cognitive stimulation, and inadequate access to clean water, improved sanitation, and quality healthcare services [10]. Research consistently shows that malnutrition during early childhood is associated with lower educational attainment and poorer performance on cognitive assessments compared to well-nourished peers [11]. Moreover, early childhood growth retardation is strongly linked to long-term functional impairments during school age and into adulthood, resulting in diminished labor productivity and reduced economic potential [12,13].

It is widely acknowledged that regular participation in physical activity (PA) is positively associated with children’s overall health and well-being. A physically active lifestyle supports the development of a strong musculoskeletal system [14], reduces the risk of chronic diseases [15], and enhances mental and cognitive functions, thereby promoting better brain health. These benefits collectively contribute to improved academic performance in both children and adolescents [16].

A growing body of research underscores the synergistic roles of PA and micronutrient intake in promoting cognitive development in children. Specifically, moderate-to-vigorous physical activity (MVPA) has been shown to improve executive functioning and accelerate cognitive processing, while higher cardiovascular fitness is associated with better memory performance and faster information processing [17,18]. PA also positively influences language development, memory, and executive function, partly through its effect on hippocampal development, which plays a critical role in memory formation.

Similarly, micronutrients are essential for healthy brain development, as several vitamins and minerals directly support the physiological processes underlying cognitive function [19,20,21]. Micronutrients such as iron, iodine, zinc, folate, vitamin B6, vitamin B12, and vitamin A are essential for brain development and contribute to improved cognitive function in children [19,21,22]. Deficiencies in these nutrients are widely associated with impaired cognitive performance, especially during childhood and adolescence, when the brain is undergoing rapid development [23]. Among these, vitamin A, iodine, and iron deficiencies are considered the most critical from a public health point of view [24], particularly in LMICs, where they significantly affect the health of young children and pregnant women [25].

Given the accelerated brain development during early and middle childhood [26], nutritional deficits during this period can lead to cognitive impairments, including reduced IQ, poor memory, difficulties in verbal and non-verbal learning, attention deficits, and slower processing speeds [27,28]. Impaired cognitive development in middle childhood is a strong predictor of later academic challenges, including lower school performance and reduced learning capacity [29,30]. Research has shown that supplementation with iron [31] and iodine [32] can enhance specific domains of cognitive development among school-age children, with particularly notable effects observed in those who are deficient in these micronutrients. Furthermore, studies have demonstrated that children with existing micronutrient deficiencies tend to show greater cognitive improvements following supplementation interventions [33,34,35].

However, some studies in high-income countries (HICs) have also reported positive cognitive effects of micronutrient supplementation among healthy children [27]. Among three studies examining the impact of supplementation on healthy participants in HICs, two demonstrated significant improvements in at least one cognitive domain following supplementation [36,37]. These findings suggest that even in resource-rich environments, children and adolescents remain at risk of micronutrient deficiencies and are particularly vulnerable to impaired cognitive development, which may have long-term consequences extending into adulthood. Hence, multi-micronutrient supplementation (MMNS) may be a valuable strategy for improving cognitive performance across multiple domains [38].

Despite the wealth of data on maternal and child nutrition [39,40], there remains a paucity of epidemiological research on cognitive and academic outcomes among school-age children in LMICs. This study aimed to evaluate the effects of school-based PA, MMNS, and PA + MMNS on cognitive function and academic achievement among primary schoolchildren in peri-urban Tanzania. As a secondary analysis of the KaziAfya trial, which primarily focused on physical health outcomes such as child health and well-being, this investigation expands the scope to examine cognitive and academic domains as key secondary endpoints. We hypothesize that MMNS will enhance academic performance through improved nutritional status, while PA will support cognitive development by stimulating executive function, attention, and neuroplasticity. Furthermore, we anticipate that the PA + MMNS will yield additive or synergistic effects on both cognitive and academic outcomes.

Recent systematic reviews support this hypothesis: Tam et al. (2020) and Roberts et al. (2022) confirm the benefits of micronutrient supplementation for cognitive function, especially in populations with existing deficiencies [41,42], while Nyaradi et al. (2021) and Donnelly et al. (2016) document improvements in executive function and academic outcomes following regular PA in school-age children [4,43]. However, most of this evidence originates from HICs, where environmental and educational conditions differ substantially from those in LMICs. In contrast, children in LMICs including Tanzania are more frequently affected by undernutrition, limited educational resources, and restricted access to structured physical activity programs, making them particularly vulnerable to poor cognitive and educational outcomes. Tanzania-specific studies have highlighted persistent challenges in child nutrition [44,45], educational attainment, and developmental outcomes [43,46], stunting and developmental delays [45], and poor academic performance linked to socio-environmental factors [46] underscoring the pressing need for scalable and context-sensitive interventions. Although PA and MMNS have individually been shown to benefit cognition, there is a significant research gap regarding their combined impact, particularly in LMIC settings. This study therefore addresses a critical gap by assessing whether integrated, school-based PA and MMNS interventions can enhance cognitive and academic outcomes in a resource-constrained context.

## 2. Materials and Methods

### 2.1. Study Design

This study draws on longitudinal data collected between 2019 and 2021 from Tan-zanian schoolchildren as part of the KaziAfya project, a cluster-randomized, placebo-controlled trial using a 2 × 2 factorial design. The trial was designed to evaluate the separate and combined effects of a school-based PA program and a daily MMNS intervention on children’s growth, health, and well-being. The KaziAfya project was implemented in three countries: Tanzania, Côte d’Ivoire, and South Africa. Based on an a priori power analysis using G*Power 3.1 [47], the study aimed to recruit 1320 schoolchildren in each country. Participants were followed over the course of two school years, with assessments conducted at three time points: baseline (T1), 14 months after baseline (T2), and 26 months after baseline (T3).

### 2.2. Participants and Procedures

Four public primary schools (i.e., Katindiuka, Kibaoni, Kining’ina, and Miembeni) were selected from a pool of 33 schools in Ifakara Town Council, located in the southeastern region of Tanzania. School selection was based on their willingness to participate and their ability to meet at least three criteria: (i) being a public institution; (ii) having adequate facilities for physical education classes; and (iii) not being involved in any other clinical trial or nutritional intervention at the time of recruitment. School principals were informed about the study’s objectives, procedures, potential risks, and expected benefits, and were given the opportunity to voluntarily express interest in participation. Children were eligible for inclusion if they (i) were enrolled in grades 1 to 4 at baseline; (ii) were aged between 6 and 12 years; (iii) had written informed consent from a parent or legal guardian; and (iv) had no medical conditions that would preclude participation in PA sessions. A more detailed description of the eligibility criteria is available in the published study protocol [47]. Parents and guardians were informed that participation in the study was voluntary, all data would be handled confidentially, and participants could withdraw at any point without further obligations. Children whose parents or guardians provided written consent were then invited to an information session, where the study’s aims, procedures, potential risks and benefits were explained in age-appropriate language. After this session, schoolchildren were asked to provide their written assent to participate in the study.

### 2.3. Interventions

Overall, 1034 schoolchildren were recruited and participated in the study. Within each school, four classes were randomly assigned to one of the three intervention groups: (i) PA + placebo; (ii) MMNS alone; (iii) PA and MMNS combined; and (iv) placebo control group. Randomization was conducted at the class level using a computer-generated randomization list to ensure allocation concealment and to prevent contamination between groups. No stratification was applied during the process. The intervention began in February 2020 with the implementation of the PA program. However, due to COVID-19-related school closures and public health restrictions, all intervention activities were suspended for approximately 6 months. PA sessions resumed in August 2020 and continued through November 2021. The distribution of MMNS supplements commenced in February 2021 and was carried out until November 2021.

#### 2.3.1. Physical Activity

Children assigned to the PA groups (alone or in combination with MMNS) received two 45 min PA classes per week. These sessions included activities such as “moving to music” and “physical education”. The intervention was based on the KaziKidz program (www.kazibantu.org, accessed on 9 August 2021), which integrates structured PA into the regular school timetable and is designed to enhance PA levels, school satisfaction, and psychosocial well-being. The frequency and duration of PA were selected to ensure compatibility with the existing school curriculum and were shown to be feasible and engaging in similar resource-limited educational settings. The KaziKidz materials had previously been pilot-tested in the DASH study (2015–2016) among fourth-grade children in disadvantaged schools in Gqeberha (formerly know as Port Elizabeth), South Africa. Results from this pilot showed strong acceptability among students and teachers, and positive effects on PA engagement and well-being. In our study, trained physical education instructors supported classroom teachers in delivering the PA lessons to ensure fidelity of implementation. Children allocated to MMNS alone or the placebo control group received the usual school lessons taught by teachers.

#### 2.3.2. Multi-Micronutrient Supplementation

Children who were assigned to the MMNS alone or the PA + MMNS groups were provided with a daily chewing tablet containing vitamins and trace elements in the morning, distributed by the teacher. The tablet was formulated using the MixMeTM powder sprinkle (DSM; Basel, Switzerland), wherein vitamin A was substituted with 4500 mg of β-carotene. Of note, β-carotene supplementation has been reported to reduce body mass index (BMI) Z-scores and abdominal adiposity in obese children [48]. The MMNS was produced and stored according to the manufacturer’s guidelines, including packaging and quality checks before delivery to the schools. Teachers administered the MMNS tablets and placebo tablets (similar in taste and appearance) five days per week to the children in the different intervention arms (Supplementary Table S1).

### 2.4. Sample Size Calculation

We determined the sample size through a priori power analyses using G*Power 3.1 (an open-source software available at https://www.psychologie.hhu.de/arbeitsgruppen/allgemeine-psychologie-und-arbeitspsychologie/gpower; accessed on 24 April 2022) [49], based on a 2 × 2 factorial design (MANOVA: repeated measure, within–between interaction). We assessed effect sizes from previous research on our proposed outcomes. MMNS interventions among preschoolers from marginalized communities showed small-to-medium–large effect sizes for inhibitory control (d = 0.2) [50]. However, the limited literature on MMNS interventions for primary schoolchildren reveals smaller and inconsistent effect sizes [50]. Research on school-based PA interventions on cognitive performance shows comparable effect sizes, with an average of d = 0.18 [51].

Given these findings, we used a conservative effect size of f = 0.1 (d = 0.2) for our sample calculation. With this effect size, an alpha error probability of 0.05, and a 0.50 correlation among repeated measures, our power analysis indicated the need for 1095 participants to achieve 80% statistical power. Accounting for an expected 10% annual dropout rate [52], the target sample size was 1320 children per country (330 per intervention arm). Despite attrition and the exclusion of cases with missing data, the final analytic sample (*n* = 559) remained adequately powered to detect small-to-moderate intervention effects in the primary outcomes.

### 2.5. Data Assessment

Data collection from children was conducted on school grounds between July and September 2019 (baseline) and September and December 2021 (post-intervention). At baseline, we collected cognitive function (Flanker task), academic achievement, anthropometric measures, SES, food insecurity, dietary diversity, and MVPA data. Parent/guardian data on food insecurity, dietary diversity, and SES were collected at participants’ household by trained research assistants at baseline. Post-intervention collection included cognitive function, academic achievement, and anthropometric measures. Baseline values served as covariates in adjusted statistical models.

### 2.6. Measures

Food insecurity was assessed with the three-item household hunger scale [53]. Dietary diversity reflected the number of the nine women’s dietary diversity score food groups consumed in the previous 24 h [54,55]. SES was derived by principal component analysis of household assets and amenities, then collapsed into tertiles (i.e., lowest, middle, and highest) [56]. Children’s hemoglobin concentration was measured capillary-wise, using a HemoCue 301 (HemoCue AB; Ängelholm, Sweden); anemia was defined as hemoglobin (Hb) < 11.5 g/dL for 5–11-year-olds and <12.0 g/dL for 12–14-year-olds [57]. Anthropometric measurements (i.e., weight and height) were converted into age- and sex-standardized Z-scores using the WHO growth reference standards. Stunting was defined as a height-for-age Z-score (HAZ) below −2 standard deviations (SD). Detailed data collection procedures are described in Minja et al. (2021) and Minja et al. (2024) [8,58].

### 2.7. Academic Achievement

School principals provided end-of-year academic records. For academic performance, we used mid-term (June) and end-of-year (December) marks for both baseline (2019) and post-intervention assessments (2020 and 2021). We measured academic achievement by averaging scores in two core subjects: home language (Kiswahili) and mathematics. The Tanzanian school system evaluates academic performance using a five-point grading scale, where higher scores reflect better achievement. Kiswahili and mathematics scores were based on students’ performance in standardized language and math tests administered as part of the national school curriculum. These scores represent the average of the mid-term and end-of-year results. The grading scale used was as follows: A = 81–100, B = 61–80, C = 41–60, D = 21–40, and F = 0–20.

### 2.8. Cognitive Function

The computerized Flanker task, which evaluates inhibitory control and information processing, was used to measure cognitive function. Assessments were conducted at baseline (T1) and the second post-intervention (T3). The E-Prime 2.0 Software (Psychology Software Tools; Sharpsburg, PA, USA) was used to program and administer the assignment. Children sat in front of computers and researchers gave instructions before the task began. Five white fish were displayed against a black screen as stimuli. Either the flanking fish or the central target fish pointed in the same direction (congruent trial) or in a different direction (incongruent trial). Children were instructed to concentrate on the center fish and press the left or right button to indicate the fish’s direction. Both accuracy and response time were given equal weight. Two practice rounds of 60 trials were followed by two blocks (30 s intervals between blocks) of 40 trials each. Congruent and incongruent trials were delivered in a random order and with equal probability. Participants had 2.5 ms to react once the visual stimulation started. The inter-trial period adjusted to decrease the probability of guessing, ranging from 1100 to 1500 ms. Performance was assessed using two key indicators: accuracy, defined as a percentage of correct responses, and reaction time, defined as the average (in ms) taken for correct responses. These indicators were calculated separately for congruent and incongruent trials. To ensure participants had understood the task, datasets with accuracy scores at or below 50% on the second practice round were excluded from analysis.

### 2.9. Statistical Analysis

All statistical analyses were conducted using the open-source software R, version 4.3.2. Descriptive characteristics of the schoolchildren at both baseline (T1) (Supplementary Table S2) and the second follow-up (T3) were summarized using means and 95% confidence intervals (CIs). A complete case analysis was conducted to handle missing data: observations with missing values in key outcome and covariate variables including height, weight, age, sex, and height-for-age Z-score (HAZ) were excluded from the analysis. This ensured that all models were based only on participants with complete data for all variables of interest at T3.

Group comparisons for categorical variables such as sex, stunting status, MVPA, and SES were performed using chi-squared (χ^2^) tests. For continuous variables (e.g., age, height, weight, BMI, zBMI, Hb concentration, Flanker task accuracy, reaction time, and academic test scores), comparisons across the four study arms (PA + placebo, MMNS, PA + MMNS, and placebo control) were conducted using one-way analyses of variance (ANOVA). Where ANOVA yielded significant results, post hoc comparisons were performed using Tukey’s Honest Significant Difference (HSD) test with Bonferroni adjustment to identify specific group differences (see Supplementary Materials).

Due to COVID-19-related school closures, the number of students assessed at T2 was insufficient, and the data were excluded from analyses to avoid bias. As such, the primary analysis focused on change from baseline (T1) to endline (T3, 26 months). Key covariates including SES, food insecurity, dietary intake (from a food frequency questionnaire), and MVPA were measured at baseline and included in all models to control for potential confounding.

To evaluate intervention effects on cognitive and academic outcomes, we used mixed-effects linear regression models, incorporating random intercepts for classes within schools to account for the hierarchical data structure (i.e., children nested within classes). 

To quantify the degree of clustering, intraclass correlation coefficients (ICCs) were calculated for each outcome variable. The ICCs reflect the proportion of variance attributable to class-level clustering and are presented in Supplementary Table S3. Intervention groups were included as fixed effects using dummy coding, with the placebo group serving as the reference category. Separate models were fit for each outcome variable at T3 (e.g., Flanker accuracy, reaction time, and academic test scores), adjusting for child-level covariates such as age, sex, zBMI, stunting status, MVPA, dietary diversity, food insecurity, SES, and the baseline value of the respective outcome. We also conducted additional models including age-by-intervention interaction terms for each outcome (see Supplementary Table S4). Regarding multicollinearity, variance inflation factors (VIFs) were calculated for all covariates using a fixed-effects linear model (see Supplementary Table S5). The results of the regression analyses are presented as regression coefficients, standard errors (SEs), and p-values.

The results of the regression analyses are presented as regression coefficients, standard errors (SEs), and *p*-values.

## 3. Results

### 3.1. Baseline Demographic and Anthropometric Characteristics of Schoolchildren

**Figure 1:**
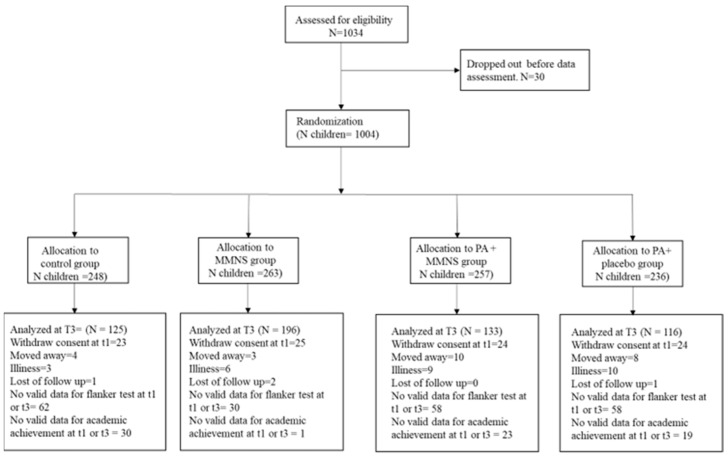
CONSORT flow diagram of children in the Tanzanian component of the KaziAfya project included in the analyses. Notes: Children could be excluded for one or more reasons (e.g., a child with a missing Flanker test could also have a missing academic achievement). PA = physical activity, MMNS = multi-micronutrient supplementation, T1 = baseline, T3 = post-intervention. The corresponding CONSORT checklist is provided in Supplementary Table S6.

A total of 1034 children aged 6–12 years were enrolled in the KaziAfya trial in Tanzania. Valid baseline and post-intervention data for cognitive performance and academic achievement, and valid age and sex data were available for 559 children, comprising 233 boys and 326 girls. A flow diagram including numbers for valid data for each group can be found in Figure 1.

Significant sex differences were observed in several anthropometric measures. Boys were significantly older than girls (10.1 years vs. 9.5 years, *p* <0.001), taller (130.8 cm vs. 128.8 cm, *p* = 0.01), and heavier (28.1 kg vs. 27.1 kg, *p* = 0.05). No significant differences were found in BMI or zBMI between boys and girls (*p* = 0.42 and *p* = 0.85, respectively).

At baseline, boys exhibited significantly faster reaction times than girls for congruent stimuli in the cognitive function task (1118 ms vs. 1173 ms, *p* = 0.01). However, no significant sex differences were observed in reaction time or accuracy for incongruent stimuli. In terms of academic achievement, girls outperformed boys in Kiswahili (68.4 vs. 61.8, *p* <0.001), while no significant differences were found in mathematics or overall end-of-year results.

Regarding dietary indicators, boys showed slightly higher dietary diversity scores than girls, though the difference was not significant (*p* = 0.30). However, girls reported significantly higher food insecurity (*p* < 0.001). Hb levels did not significantly differ between boys and girls. The prevalence of stunting was similar across genders (*p* = 0.27). Notably, a significantly higher proportion of girls met the recommended levels for MVPA compared to boys (58.0% vs. 42.0%, *p* = 0.01). SES was comparable for boys and girls across low, middle, and high categories, with no significant group differences observed (*p* = 0.39) (Table 1).

At T3, the prevalence of stunting was comparable across groups (*p* = 0.61), ranging from 23.5% in the MMNS group to 27.8% in the PA + MMNS group. The effect size was small (0.18, 95% CI: −0.37 to 0.74), indicating minimal practical difference between groups. The mean age significantly differed between groups (*p* < 0.001), with the PA + MMNS group being the oldest (12.4 years) and the placebo group the youngest (11.1 years). No significant differences were found in height (*p* = 0.12) and weight (*p* = 0.25); however, BMI was significantly different across groups (*p* = 0.02), with the PA + MMNS group having the highest BMI (18.2 kg/m^2^) (Table 2).

#### 3.1.1 Cognitive Function

**Figure 2:**
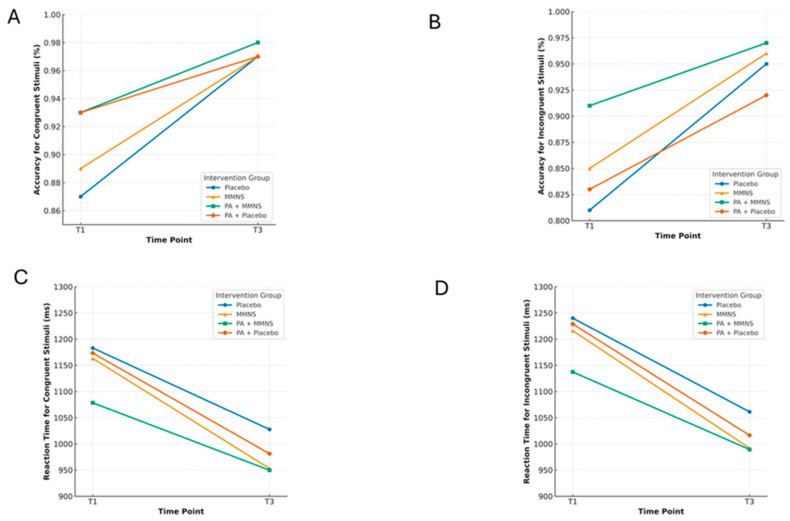
Accuracy and reaction time on the Flanker task displayed for T1 and T3. Notes. (**A**) = accuracy (congruent), (**B**) = accuracy (incongruent), (**C**) = reaction time (congruent), and (**D**) = reaction time (incongruent) on the Flanker task displayed for each intervention group at baseline (T1) and after intervention (T3). See Supplementary Figure S1 for Cohen’s *d* effect sizes of within-group changes from baseline (T1) to post-intervention (T3) across four cognitive outcomes.

In terms of cognitive function, accuracy on congruent stimuli did not differ significantly between groups (*p* = 0.15). However, accuracy for incongruent stimuli was significantly different (*p* < 0.001), with the PA + MMNS group showing the highest accuracy (0.97), and the PA + placebo group the lowest (0.91). Reaction time for congruent stimuli was also significantly different (*p* = 0.03), with the PA + MMNS group responding the fastest (956 ms), while the placebo group had the slowest responses (1028 ms). No significant group differences were observed for reaction time for incongruent stimuli (*p* = 0.11) (Table 2 and Figure 2).

#### 3.1.2 Academic Achievement

Regarding academic achievement, end-of-year results significantly differed between groups (*p* < 0.001), with the placebo group scoring highest (M = 313.3, 95% CI: 307.0–319.0) and the PA + placebo group lowest (M = 271.2, 95% CI: 263.0–279.0). Kiswahili performance also differed significantly (*p* < 0.001), with the MMNS group showing the highest scores (M = 58.0, 95% CI: 56.6–59.4). Mathematics scores varied significantly across groups (*p* < 0.001), with the placebo group again outperforming the others (Table 2 and Figure 2).

### 3.2. Exploratory Post Hoc Analyses

In the Bonferroni post hoc comparisons, several significant differences were observed between the intervention groups for various covariates at T3. For age, significant differences were found between the placebo and PA + MMNS groups (*p* < 0.01), and between MMNS and PA + MMNS (*p* < 0.01). Regarding BMI, a significant difference was noted between the placebo and PA + MMNS groups (*p* = 0.02). For accuracy in the incongruent stimuli condition, significant differences were observed between the placebo and MMNS vs. PA (*p* = 0.01) and PA + MMNS vs. PA and placebo + PA (*p* < 0.01). In academic outcomes, Kiswahili results showed significant differences between placebo and MMNS (*p* < 0.01), MMNS and PA + MMNS (*p* < 0.01), and MMNS and PA (*p* < 0.01). Mathematics showed significant differences between placebo and MMNS (*p* < 0.01), and placebo and PA + MMNS (*p* < 0.01). These results suggest that certain interventions may have led to significant improvements in outcomes compared to placebo, especially in terms of accuracy and academic performance (Supplementary Materials, Table S7).

### 3.3. Mixed Multiple Linear Regression Analyses

#### 3.3.1. Unadjusted Model

Unadjusted mixed multiple linear regression analyses indicated that MMNS was associated with significantly higher end-of-year academic results and Kiswahili performance compared to placebo. PA+MMNS also showed significantly higher Kiswahili performance, while PA alone was associated with lower accuracy on incongruent stimuli; no other significant effects were observed for cognitive outcomes (Supplementary Materials, Table S8).

#### 3.3.2. Adjusted Model

##### Cognitive Function

Compared to the placebo group, no significant effects of the MMNS, PA, or the combined PA + MMNS interventions were observed on accuracy for either congruent or incongruent stimuli. However, sex was significantly associated with performance (*p* = 0.01), suggesting lower accuracy among boys. Hb level was positively associated with accuracy (*p* < 0.001) and baseline accuracy for congruent stimuli positively predicted post-intervention accuracy for congruent stimuli (*p* < 0.001) (Table 3). Additionally, age showed a significant negative association (*p* = 0.01), and baseline accuracy remained a significant predictor (*p* < 0.001) of accuracy for incongruent stimuli.

The intervention showed no significant effect on reaction time for either congruent or incongruent stimuli when compared to the placebo group. However, sex turned out to be a significant predictor in both conditions, with boys responding significantly faster than girls (*p* < 0.001). Additionally, baseline reaction time predicted post-intervention performance in both congruent and incongruent tasks (*p* < 0.001).

##### Academic Achievement

The PA intervention was significantly associated with lower end-of-year academic scores (*p* < 0.001) compared to the placebo group. No significant effects were observed for MMNS or PA + MMNS groups compared to placebo groups. Baseline assessments significantly predicted the scores at T3 (*p* < 0.001). For performance in Kiswahili, the MMNS intervention led to significant improvements (*p* < 0.001), while the PA + MMNS group also showed a significant positive effect (*p* = 0.01), whereas PA + placebo showed no significant association. Baseline Kiswahili performance was a strong predictor of performance in this subject at T3 (*p* < 0.001). No other variables were significant predictors. None of the intervention groups showed significant effects on the performance in mathematics. However, sex was a significant predictor, with boys scoring higher than girls (*p* = 0.04), and age was negatively associated with performance in mathematics (*p* = 0.03). Baseline mathematics scores were a strong predictor of post-intervention outcomes (*p* < 0.001).

### 3.4. Summary of Key Results

Overall, children in the placebo group consistently outperformed those in intervention groups across multiple academic outcomes, particularly in end-of-year results and mathematics. The PA + placebo intervention was associated with significantly lower academic scores compared to the placebo group. In contrast, the MMNS intervention led to significant improvements in Kiswahili performance, and the combined PA + MMNS group also showed a positive effect, though less pronounced. Post hoc analyses confirmed these findings, showing significant differences between groups, especially for Kiswahili performance, where the MMNS group outperformed both the PA and PA + MMNS groups. For cognitive outcomes, the PA + MMNS group showed the highest accuracy on incongruent stimuli, though mixed-model analysis did not show statistically significant intervention effects after adjustment. Regarding sex and age, girls performed significantly better in Kiswahili, while boys had faster reaction times in the Flanker task. Older children tended to have lower cognitive and academic scores, indicating an inverse association with age in some outcomes. These patterns suggest the need to consider baseline differences and demographic effects when interpreting intervention outcomes.

## 4. Discussion

The key findings of our study can be summarized as follows: (1) MMNS and PA + MMNS interventions significantly improved performance in Kiswahili, but not mathematics; (2) the PA-only intervention was associated with significantly lower end-of-year academic scores; and (3) none of the interventions significantly affected cognitive performance in terms of Flanker task accuracy or reaction time. However, individual characteristics such as sex, age, Hb levels, and baseline scores were significant predictors of both academic and cognitive outcomes. These results highlight domain-specific intervention effects and underscore the importance of contextual and individual factors in interpreting school-based intervention outcomes.

### 4.1. Effect of the MMNS Intervention

MMNS is widely regarded as a key strategy for improving cognitive outcomes, particularly in regions with high levels of undernutrition and micronutrient deficiencies [42]. Our study was conducted in the Kilombero district, an area with documented deficiencies in essential nutrients such as iron and vitamin B [8,45], both critical to cognitive development. Despite these conditions and the implementation of MMNS, our findings revealed no significant improvements in cognitive function (accuracy or reaction time) among children in the MMNS group compared to placebo recipients. However, MMNS was associated with improved academic performance, particularly in language-related outcomes such as Kiswahili. This aligns with prior research suggesting that adequate micronutrient status can support better academic achievement [38] and cognitive function [42], possibly due to enhanced attention and learning capacity. The lack of impact on cognitive task performance may indicate that MMNS has more pronounced effects on sustained academic engagement than on short-term neurocognitive responses. Notably, the MMNS group also recorded the lowest prevalence of stunting (23.5%) among the intervention arms, suggesting some nutritional benefit. While differences in height and weight were not statistically significant, this lower stunting rate may reflect improved nutritional status, potentially contributing to better school performance. The literature on MMNS and cognitive outcomes presents mixed findings. For instance, a South African school-based intervention reported no significant benefits for cognition or academics [59], while an 11-week MMNS program among preschoolers showed significant cognitive improvements on the Kaufman Assessment Battery [60]. Other studies have shown that the impact of MMNS can depend heavily on contextual factors, such as intervention duration, age of participants, and the quality of the educational environment [61]. Several plausible factors may explain the absence of cognitive effects in our study. First, the Flanker task included congruent trials that demand minimal inhibitory control, resulting in high accuracy scores across all groups and likely contributing to a ceiling effect [4,62]. This may have limited our ability to detect subtle improvements. Second, participants received training prior to testing, which may have reduced performance variability and further obscured differences between groups [63,64]. Third, the MMNS intervention lasted only 6 months, with a 6-week school break during which supplementation was paused, which is potentially too short to yield measurable cognitive benefits. Additionally, variation in nutrient absorption and individual baseline deficiencies may have influenced outcomes. Together, these findings suggest that while MMNS may enhance academic performance, particularly in language, it may not produce immediate improvements in standardized cognitive tasks, especially those prone to ceiling effects or influenced by training.

### 4.2. Effect of the PA + Placebo Intervention

It was initially hypothesized that the PA intervention would enhance cognitive function and academic achievement. However, contrary to expectations, our findings indicate that children in the PA group had lower accuracy on incongruent stimuli and significantly lower end-of-year academic scores compared to the placebo group and other intervention arms. These findings contrast with a study conducted among South African schoolchildren, where no additional cognitive benefits were observed from the PA intervention, as all groups exhibited cognitive improvement [59]. Furthermore, a school-based PA program implemented among older South African primary schoolchildren reported no significant improvements or declines in selective attention and academic achievement when compared to children following a regular academic curriculum [65]. These differences indicate that the effectiveness of PA interventions on cognitive and academic performance may vary depending on context, with key moderating factors including intervention intensity, duration, integration with academic instruction, baseline cognitive abilities, and adequate nutrition, all of which can influence different cognitive domains. One possible concern in school-based PA research is that adding PA may disrupt academic instruction time. However, we clarify that in our study, PA sessions were scheduled during existing free periods in the school timetable in coordination with school administrators. These sessions did not replace core academic subjects. Thus, the observed lower academic performance in the PA group is unlikely to be explained by lost instructional time. Instead, other contextual factors may have contributed to these findings.

Deficiencies in essential micronutrients such as iron, zinc, and vitamin B12, which play a critical role in cognitive function [66], may have exacerbated the negative effects of PA on cognitive tasks requiring attention and inhibitory control. Moreover, not all PA activities provide equal cognitive benefits; activities that involve coordination, strategic thinking, and problem solving (e.g., strategy-based games, complex movement patterns, and team sports) are generally more effective in enhancing cognitive function [67] than repetitive aerobic exercises. Engaging in activities that require continuous cognitive input allows children and adolescents to practice self-control, goal-directed behavior, and cognitive flexibility [68]. In the present study, the PA intervention incorporated a variety of exercise types, including locomotor skills, coordination activities, sports, and games. However, the frequency and intensity of these activities may have been insufficient to yield significant cognitive and academic benefits. Additionally, large class sizes and a limited number of trained sports teachers may have further restricted the intensity and quality of physical education lessons [65]. The lack of qualified physical education teachers and structured sports classes in Tanzanian public primary schools remains a significant barrier that must be addressed by the educational system to optimize the cognitive and academic benefits of school-based PA interventions.

### 4.3. Effect of the Combined PA and MMNS Intervention

It was hypothesized that the combined PA + MMNS intervention would yield greater benefits than either intervention alone or the placebo, particularly in enhancing cognitive function and academic achievement. However, in our study, the PA + MMNS group did not show significant improvements in accuracy for either congruent or incongruent stimuli, nor in reaction time. These findings suggest that the combined intervention did not produce measurable gains in cognitive control or information processing speed. Notably, the PA + MMNS group showed a positive effect on academic performance, particularly in Kiswahili scores. This contrasts with a randomized controlled trial (RCT) in South African primary schools, where all intervention groups including the combined PA and MMNS group demonstrated cognitive gains [59]. One possible reason for the lack of cognitive improvements in our study may relate to the duration and intensity of the intervention. The MMNS lasted only 6 months, with a mid-term break during which supplementation was stopped. This may have been insufficient to produce detectable changes in cognitive function, especially in a population with a high prevalence of chronic micronutrient deficiencies. Additionally, the dosage or bioavailability of the supplement may not have been optimal for eliciting measurable neurocognitive effects. In contrast, the observed academic improvements, particularly in Kiswahili, may reflect indirect benefits of the combined intervention. These could include enhanced classroom engagement, improved overall well-being, or increased energy levels, all of which support academic learning. It is important to note that the PA + MMNS group did not lose instructional time, as PA sessions were scheduled during existing free periods in collaboration with school staff. Thus, the positive academic effects are unlikely to have come at the cost of reduced teaching hours. The association between the combined intervention and Kiswahili performance may also be influenced by contextual factors such as language exposure, teaching quality, or home literacy environments. The lack of a similar effect in mathematics suggests that different academic domains may respond differently to school-based interventions. Moreover, as with the other outcomes, performance was shaped by baseline scores, sex, age, and dietary diversity, indicating that multiple factors beyond the intervention contribute to educational outcomes. These findings underscore the complexity of influencing academic achievement and highlight the need for multi-dimensional, context-sensitive approaches [69].

### 4.4. Strengths and Limitations

We would like to highlight several strengths of our study. First, the randomized factorial design allowed us to independently assess the effects of PA + placebo, MMNS, and their combination on cognitive and academic outcomes, enhancing the internal validity of our findings. To our knowledge, this is the first RCT to evaluate the combined impact of PA and MMNS on schoolchildren’s health and development in a peri-urban Tanzanian context, helping to fill a critical geographic and evidence gap. Second, the study was implemented in resource constrained public primary schools, offering valuable insights into the feasibility and effectiveness of school-based health interventions in low-income settings. Third, cognitive function was measured using scientifically validated and globally recognized tools (e.g., the Flanker task), ensuring the reliability of assessments. Fourth, adherence to the supplementation protocol was closely monitored, with placebo tablets matched in appearance and taste to ensure blinding. Lastly, our inclusion of relevant covariates such as Hb concentration and household food insecurity enabled a more comprehensive analysis of the factors influencing children’s cognitive and academic outcomes.

We acknowledge that this study has several limitations. First, the MMNS intervention lasted only 6 months, which may have been insufficient to appreciate its full effects, particularly given the high prevalence of undernutrition and micronutrient deficiencies in the study population. A longer supplementation period might be required to detect significant cognitive benefits.

Second, limited access to computers at home among the participating children may have disadvantaged some during the computerized cognitive function task (Flanker task), despite the inclusion of two practice rounds before testing. In addition, the simplicity of the congruent trials likely contributed to high accuracy scores across all groups, suggesting a potential ceiling effect that may have masked subtle intervention-related improvements. We further acknowledge that future studies might consider alternative or complementary tools, such as the Stroop task, which may provide greater sensitivity for capturing subtle cognitive changes related to nutrition and PA interventions.

Third, the study’s findings may not be generalizable to children from other LMICs, different geographic settings, or varying socioeconomic backgrounds, as the study was conducted specifically in peri-urban areas of Tanzania. Fourth, while clinical examinations were conducted prior to the intervention, children were not screened for neurological disorders, such as attention deficit hyperactivity disorder (ADHD) or fetal alcohol syndrome, both of which are associated with cognitive impairments and could have influenced the results. Fifth, there is a possibility that children in the placebo group also became more physically active during school hours, as PA sessions were conducted outdoors, potentially leading to an unintended increase in their activity levels. Lastly, the exclusion of T2 data from the final analysis may have affected the statistical power and interpretation of results. The reduced number of measurement points may have limited our ability to detect more nuanced intervention effects over time. In addition, our reliance on complete case analysis may have introduced bias if the excluded data had not been absent in a completely random way, and it reduced the overall sample size, potentially affecting the generalizability of the findings.

## 5. Conclusions

The findings from this study, conducted in peri-urban Tanzania, suggest that while the PA intervention was associated with lower end-of-year academic scores, the MMNS intervention led to significant improvements in Kiswahili performance. The combined PA + MMNS intervention also demonstrated positive effects on language outcomes. No significant intervention effects were observed for mathematics performance. Baseline academic scores in both Kiswahili and mathematics emerged as strong predictors of post-intervention achievement, highlighting the importance of initial academic levels. Additionally, individual characteristics including sex, age, and Hb levels were significantly associated with academic and cognitive outcomes. For example, boys showed faster reaction times, but lower accuracy compared to girls, while higher Hb levels were linked to better accuracy. However, none of the interventions significantly improved accuracy or reaction time in the Flanker task, suggesting limited short-term cognitive benefits. These findings may be partly explained by potential ceiling effects and the relatively short duration of the intervention. Overall, while MMNS may support language-related academic outcomes, its impact on broader cognitive function and mathematics performance remains inconclusive. Future studies in similar low-resource settings should consider longer supplementation periods, more cognitively demanding assessments (e.g., the Stroop task), and multi-level strategies addressing both educational and health-related factors. Findings should be interpreted within the specific context of peri-urban Tanzania and may not be generalizable to other settings.

## Figures and Tables

**Table 1:** Descriptive statistics at T1 and differences between boys and girls, based on χ^2^-tests (categorical variables) and ANOVAs (continuous variables).

Measures	N	All Children (*n* = 559)	Boys (*n* = 233)	Girls (*n* = 326)	χ^2^/F Score	*p*-Value	Effect Size
		M (95% CI)	M (95% CI)	M (95% CI)			
Age (years)	570	9.8 (9.7, 9.9)	10.1 (9.9, 10.3)	9.5 (9.4, 9.7)	−4.29	<0.01	0.58 (0.32, 0.84)
Height (cm)	547	129.6 (128.8, 130.5)	130.9 (129.6, 132.2)	128.8 (127.7, 129.9)	−2.38	0.01	2.07 (0.37, 3.78)
Weight (kg)	547	27.5 (27.0, 28.1)	28.1 (27.3, 28.9)	27.1 (26.4, 27.8)	−1.89	0.06	1.01 (−0.04, 2.06)
BMI (kg/m^2^)	570	11.5 (9.6, 13.4)	12.3 (9.6, 15.0)	10.9 (8.2, 13.5)	−0.75	0.45	1.44 (−2.39, 5.30)
zBMI	547	−0.33 (−0.42, −0.24)	−0.34 (−0.47, −0.21)	−0.32 (−0.44, −0.21)	0.24	0.81	−0.02 (−0.19, 0.15)
Cognitive function							
Accuracy (congruent stimuli)	570	0.90 (0.89, 0.92)	0.91 (0.89, 0.93)	0.90 (0.88, 0.92)	−1.35	0.17	0.01 (−0.00, 0.03)
Accuracy (incongruent stimuli)	570	0.85 (0.83, 0.87)	0.86 (0.84, 0.89)	0.83 (0.81, 0.86)	−1.64	0.09	0.03 (−0.00, 0.06)
Reaction time (congruent stimuli)	570	1146 (1125, 1168)	1110 (1077, 1142)	1173 (1145, 1201)	2.88	0.01	−63.03 (−105.95, −20.11)
Reaction time (incongruent stimuli)	570	1201 (1178, 1224)	1173 (1137, 1209)	1221 (1191, 1251)	1.99	0.05	−47.87 (−94.76, −0.99)
Academic achievement							
End-of-year results	555	59.8 (57.9, 61.7)	58.6 (55.4, 61.9)	60.6 (58.3, 62.9)	0.98	0.32	−1.98 (−5.83, 1.86)
Kiswahili language	555	66.2 (64.0, 68.4)	63.2 (59.4, 67.0)	68.4 (65.7, 71.1)	2.19	<0.01	−5.21 (−9.73, −0.68)
Mathematics	555	53.4 (51.3, 55.5)	54.1 (50.7, 57.5)	52.9 (50.3, 55.5)	−0.57	0.56	1.23 (−2.94, 5.41)
Dietary							
Dietary diversity (WDDS)	532	2.92 (2.85, 2.98)	2.96 (2.85, 3.08)	2.89 (2.80, 2.97)	−1.03	0.30	0.07 (−0.06, 0.22)
Food insecurity (HHS)	532	1.30 (1.23, 1.36)	1.20 (1.11, 1.29)	1.37 (1.27, 1.46)	2.51	0.01	0.08 (−0.22, 0.40)
Nutrition							
Hemoglobin level (g/dL)	546	12.79 (12.70, 12.88)	12.85 (12.71, 12.99)	12.74 (12.62, 12.86)	−1.14	0.25	0.11 (−0.08, 0.30)
Stunting	547	119 (22)	53 (45)	66 (55)	0.25	0.61	0.12 (−0.28, 0.54)
Meeting MVPA recommendation	506	464 (92)	195 (42)	269 (58)	5.96	0.01	0.97 (0.22, 1.74)
SES							
Low	570	179 (32)	80 (45)	99 (55)	2.08	0.35	−0.25 (−0.62, 0.10)
Middle		197 (35)	85 (43)	112 (57)			
High		186 (33)	70 (38)	116 (62)			

Notes. M = mean, BMI = body mass index, CI = confidence interval, Hb = hemoglobin, HHS = household hunger scale, MVPA = moderate-to-vigorous physical activity, SES = socioeconomic status, stunting = height-for-age Z-score, WDDS = women’s dietary diversity score, zBMI = standardized BMI, χ^2^ = chi-squared value was used to compare for all categorical variables between the intervention and F-score from ANOVA was used to compare continuous variables across the intervention groups.

**Table 2:** Descriptive statistics at T3 and differences between the intervention groups and placebo control group, based on χ^2^-tests (categorical variables) and ANOVA (continuous variables).

Child Characteristics	Interventions						
	Placebo M (95% CI)	MMNS M (95% CI)	PA + MMNS M (95% CI)	PA + Placebo M (95% CI)	χ^2^/F Score	*p*-Value	Effect Size
Female, n (%)	75 (60.0%)	95 (49.2%)	86 (66.2%)	71 (62.3%)			
Male, n (%)	50 (40.0%)	98 (50.8%)	44 (33.9%)	43 (37.7%)			
Stunting	33 (26.4%)	46 (23.5%)	37 (27.8%)	32 (27.6%)	1.90	0.59	0.18 (−0.37, 0.74)
Age (years)	11.3 (11.2, 11.4)	11.7 (11.5, 11.8)	12.4 (12.3, 12.5)	11.8 (11.6, 11.9)	10.68	<0.01	1.06 (0.69, 1.44)
Height (cm)	128.4 (125.1, 133.0)	134.4 (131.0, 137.0)	121.9 (116.0, 128.0)	128.0 (124.0, 132.0)	1.69	0.16	−6.50 (−18.56, 5.56)
Weight (kg)	28.1 (26.1, 30.2)	31.9 (30.1, 33.6)	25.9 (22.6, 29.1)	28.9 (26.6, 31.3)	1.21	0.30	−2.23 (−9.21, 4.75)
BMI (kg/m^2^)	17.2 (17.0, 17.4)	17.6 (17.4, 17.8)	18.2 (18.0, 18.5)	17.9 (17.8, 18.1)	3.23	0.02	1.02 (0.33, 1.71)
zBMI	−0.31 (−0.40, −0.21)	−0.18 (−0.27, −0.10)	−0.16 (−0.25, −0.07)	−0.03 (−0.11, 0.04)	1.31	0.27	0.14 (−0.11, 0.40)
Cognitive function							
Accuracy (congruent stimuli)	0.97 (0.97, 0.98)	0.97 (0.96, 0.97)	0.98 (0.98, 0.99)	0.97 (0.96, 0.97)	1.70	0.16	0.00 (−0.00, 0.02)
Accuracy (incongruent stimuli)	0.95 (0.95, 0.96)	0.96 (0.95, 0.97)	0.97 (0.97, 0.98)	0.92 (0.91, 0.94)	4.61	<0.01	0.02 (−0.00, 0.04)
Reaction time (congruent stimuli)	1028 (1008, 1048)	953 (934, 972)	950 (934, 966)	981 (960, 1002)	3.27	0.02	−77.87 (−134.77, −20.96)
Reaction time (incongruent stimuli)	1061 (104, 1082)	992 (971, 1013)	989 (971, 1007)	1016 (995, 1038)	2.46	0.06	−71.95 (−132.68, −11.22)
Academic achievement							
End-of-year results	313.3 (307.0, 319.0)	281.6 (275.0, 288.0)	291.3 (285.0, 298.0)	271.2 (263.0, 279.0)	5.48	<0.01	−21.96 (−42.99, −0.93)
Kiswahili language	48.9 (47.7, 50.0)	58.0 (56.6, 59.4)	49.0 (47.1, 49.9)	47.6 (46.1, 49.1)	13.25	<0.01	−0.37 (−4.64, 3.88)
Mathematics	40.4 (38.9, 41.9)	31.9 (30.4, 33.5)	30.0 (29.2, 30.9)	35.2 (33.7, 36.7)	7.13	<0.01	−10.36 (−15.23, −5.49)

Notes. PA = physical activity, MMNS = multi-micronutrient supplementation, M = mean, BMI = body mass index, zBMI = BMI-for-age Z-score, CI = confidence interval, χ^2^ = chi-squared value was used to compare for all categorical variables between the intervention and F-score from ANOVA was used to compare continuous variables across the intervention groups.

**Table 3:** Mixed multiple linear regression analyses, adjusted for potential confounders, to explain the effect of the three intervention conditions on cognitive function and academic achievement, in comparison to the placebo control group.

Explanatory Variables	Mixed Multiple Linear Regression		
	Adjusted		
	Estimate	SE	*p*-Value
Accuracy at T3 (congruent stimuli)			
MMNS	−0.00	0.00	0.49
PA + placebo	−0.00	0.00	0.09
PA + MMNS	0.00	0.00	0.95
Sex (0 = girls, 1 = boys)	0.00	0.00	0.01
Age (years)	−0.00	0.00	0.06
zBMI	0.00	0.00	0.73
Stunting (0 = not stunted, 1 = stunted)	−0.01	0.00	0.12
MVPA (0 = not met, 1 = met)	0.00	0.00	0.76
Low SES	−0.00	0.00	0.05
Middle SES	−0.00	0.00	0.17
Hemoglobin	0.01	0.00	<0.01
Dietary diversity (WDDS)	−0.00	0.00	0.42
Food security (HHS)	−0.00	0.00	0.68
Baseline accuracy (congruent stimuli)	0.00	0.00	<0.01
Accuracy at T3 (incongruent stimuli)			
MMNS	−0.00	0.01	0.95
PA + placebo	−0.02	0.01	0.08
PA + MMNS	0.00	0.01	0.49
Sex	0.00	0.00	0.46
Age (years)	0.00	0.00	0.01
zBMI	−0.00	0.04	0.22
Stunting (0 = not stunted, 1 = stunted)	−0.01	0.01	0.19
MVPA (0 = not met, 1 = met)	−0.00	0.01	0.80
Low SES	−0.01	0.01	0.08
Middle SES	−0.01	0.01	0.05
Hemoglobin	0.00	0.01	0.57
Dietary diversity (WDDS)	−0.00	0.00	0.25
Food security (HHS)	0.00	0.01	0.92
Baseline accuracy (incongruent stimuli)	0.20	0.02	<0.01
Reaction time at T3 (congruent stimuli)			
MMNS	−14.17	31.74	0.65
PA + placebo	−24.07	30.75	0.43
PA + MMNS	17.95	34.78	0.61
Sex	−106.33	20.91	<0.01
Age (years)	−9.14	9.62	0.34
zBMI	0.89	9.52	0.92
Stunting (0 = not stunted, 1 = stunted)	13.95	23.80	0.55
MVPA (0 = not met, 1 = met)	32.07	37.09	0.38
Low SES	10.63	23.88	0.65
Middle SES	−4.75	23.53	0.84
Hemoglobin	−2.18	8.97	0.80
Dietary diversity (WDDS)	−10.18	11.72	0.38
Food security (HHS)	−13.19	12.81	0.30
Baseline reaction time (congruent stimuli)	0.23	0.04	<0.01
Reaction time at T3 (incongruent stimuli)			
MMNS	−14.93	34.29	0.66
PA + placebo	−17.00	33.17	0.60
PA + MMNS	11.31	37.48	0.76
Sex	−117.81	22.34	<0.01
Age (years)	−5.08	10.43	0.63
zBMI	1.55	10.30	0.88
Stunting (0 = not stunted, 1 = stunted)	7.39	25.71	0.77
MVPA (0 = not met, 1 = met)	23.97	40.02	0.55
Low SES	18.49	25.81	0.47
Middle SES	−1.38	25.45	0.95
Hemoglobin	1.15	9.66	0.90
Dietary diversity (WDDS)	−16.41	12.69	0.19
Food security (HHS)	−11.18	13.84	0.42
Baseline reaction time (incongruent stimuli)	0.21	0.03	<0.01
End-of-year results at T3			
MMNS	8.75	1.02	0.39
PA + placebo	−4.51	9.87	<0.01
PA + MMNS	2.00	1.11	0.07
Sex	1.22	6.49	0.06
Age (years)	−4.65	3.16	0.14
zBMI	7.86	3.02	0.79
Stunting (0 = not stunted, 1 = stunted)	8.08	7.59	0.28
MVPA (0 = not meet, 1 = meet)	1.02	1.13	0.36
Low SES	−3.91	7.49	0.25
Middle SES	−8.29	7.31	0.25
Hemoglobin	−2.49	2.84	0.38
Dietary diversity (WDDS)	0.00	3.85	0.99
Food security (HHS)	1.20	4.03	0.76
Baseline end-of-year results	2.05	0.14	<0.01
Performance in Kiswahili at T3			
MMNS	12.23	2.10	<0.01
PA + placebo	−3.88	2.02	0.05
PA + MMNS	7.00	2.24	0.01
Sex	0.76	1.34	0.56
Age (years)	−0.74	0.63	0.23
zBMI	0.50	0.62	0.41
Stunting (0 = not stunted, 1 = stunted)	−0.01	1.56	0.99
MVPA (0 = not met, 1 = met)	0.79	2.32	0.73
Low SES	0.37	1.55	0.80
Middle SES	−2.28	1.50	0.12
Hemoglobin	−1.05	0.58	0.07
Dietary diversity (WDDS)	−0.16	0.79	0.83
Food security (HHS)	−0.14	0.83	0.86
Baseline performance in Kiswahili	0.35	0.02	<0.01
Performance in mathematics at T3			
MMNS	−2.73	2.35	0.24
PA + placebo	0.90	2.10	0.66
PA + MMNS	0.63	2.84	0.82
Sex	2.73	1.33	0.04
Age (years)	−1.39	0.65	0.03
zBMI	−0.45	0.63	0.47
Stunting (0 = not stunted, 1 = stunted)	−1.10	1.69	0.51
MVPA (0 = not met, 1 = met)	3.06	2.66	0.25
Low SES	−0.59	1.59	0.71
Middle SES	−0.53	1.54	0.73
Hemoglobin	−0.01	0.62	0.98
Dietary diversity (WDDS)	0.66	0.81	0.41
Food security (HHS)	−0.02	0.83	0.97
Baseline performance in mathematics	0.29	0.02	<0.01

Notes: PA = physical activity, MMNS = multi-micronutrient supplementation, SE = standard error. All estimates are from linear mixed models, including group (intervention and placebo) as fixed effects, and school classes as random effects. SES = socioeconomic status, stunting = height-for-age Z-score, MVPA = moderate-to-vigorous physical activity, WDDS = women’s dietary diversity score, HHS = household hunger scale, and baseline measures of key predictors of academic achievement and cognitive outcomes function as covariates.

## Data Availability

All data for this study will be made available by the corresponding author (e-mail address: eminja@ihi.or.tz or elihaikaminja@swisstph.ch) upon reasonable request.

## Supplementary Materials

The following supporting information can be downloaded at: https://www.mdpi.com/article/10.3390/ijerph22091335/s1, Figure S1: Cohen’s d effect sizes for within-group changes from baseline (T1) to post-intervention (T3) across four cognitive outcomes. Table S1: Content of the multi-micronutrient supplementation chewing tablets. Table S2: Descriptive characteristics and baseline differences between groups. Table S3: Intraclass Correlation Coefficients (ICC). Table S4: Mixed multiple linear regression analyses, assessing age interaction. Table S5: Variance Inflation Factors (VIFs) for Covariates. Table S6: CONSORT-Checklist. Table S7: Pairwise comparisons of covariates at time point 3 between intervention groups. Table S8: unadjusted mixed multiple linear regression analyses.

## Abbreviations

The following abbreviations are used in this manuscript:

ANOVA	Analysis of variance
ADHD	Attention deficit hyperactivity disorder
BAZ	Body mass index-for-age Z-score
BIA	Bioelectrical impedance analysis
BMI	Body mass index
CI	Confidence interval
CONSORT	Consolidated Standards of Reporting Trials
COVID-19	Coronavirus disease 2019
DSM	DSM N.V. (Dutch multinational company that manufactures the MixMeTM supplement)
GEE	Generalized estimating equation
HAZ	Height-for-age Z-score
Hb	Hemoglobin
HHS	Household hunger scale
ISRCTN	International Standard Randomised Controlled Trial Number (registry)
LMICs	Low- and middle-income countries
MMNS	Multi-micronutrient supplementation
MRCC	Medical research coordinating committee
MVPA	Moderate-to-vigorous physical activity
NIMR	National Institute for Medical Research
PA	Physical activity
PA + MMNS	Combined physical activity and multi-micronutrient supplementation intervention
RCT	Randomized controlled trial
SD	Standard deviation
SES	Socioeconomic status
UNESCO	United Nations Educational, Scientific and Cultural Organization
WDDS	Women’s dietary diversity score
WHO	World Health Organization
zBMI	Age- and sex-standardized BMI Z-score
