# Supplementary material for: Effect of a School-Based Physical Activity and Multi-Micronutrient Supplementation Intervention on Cognitive Function and Academic Achievement Among Schoolchildren in Tanzania: Secondary Outcome from the KaziAfya Cluster-Randomized Controlled Trial
Source: Int J Environ Res Public Health. 2025 Aug 27;22(9):1335. doi: 10.3390/ijerph22091335 (PMC12469510; doi:10.3390/ijerph22091335)
Supplement: Supplementary file 1 [file ijerph-22-01335-s001.zip › ijerph-3702980-supplementary/Figure S1.docx_Cohens effect size.pdf]

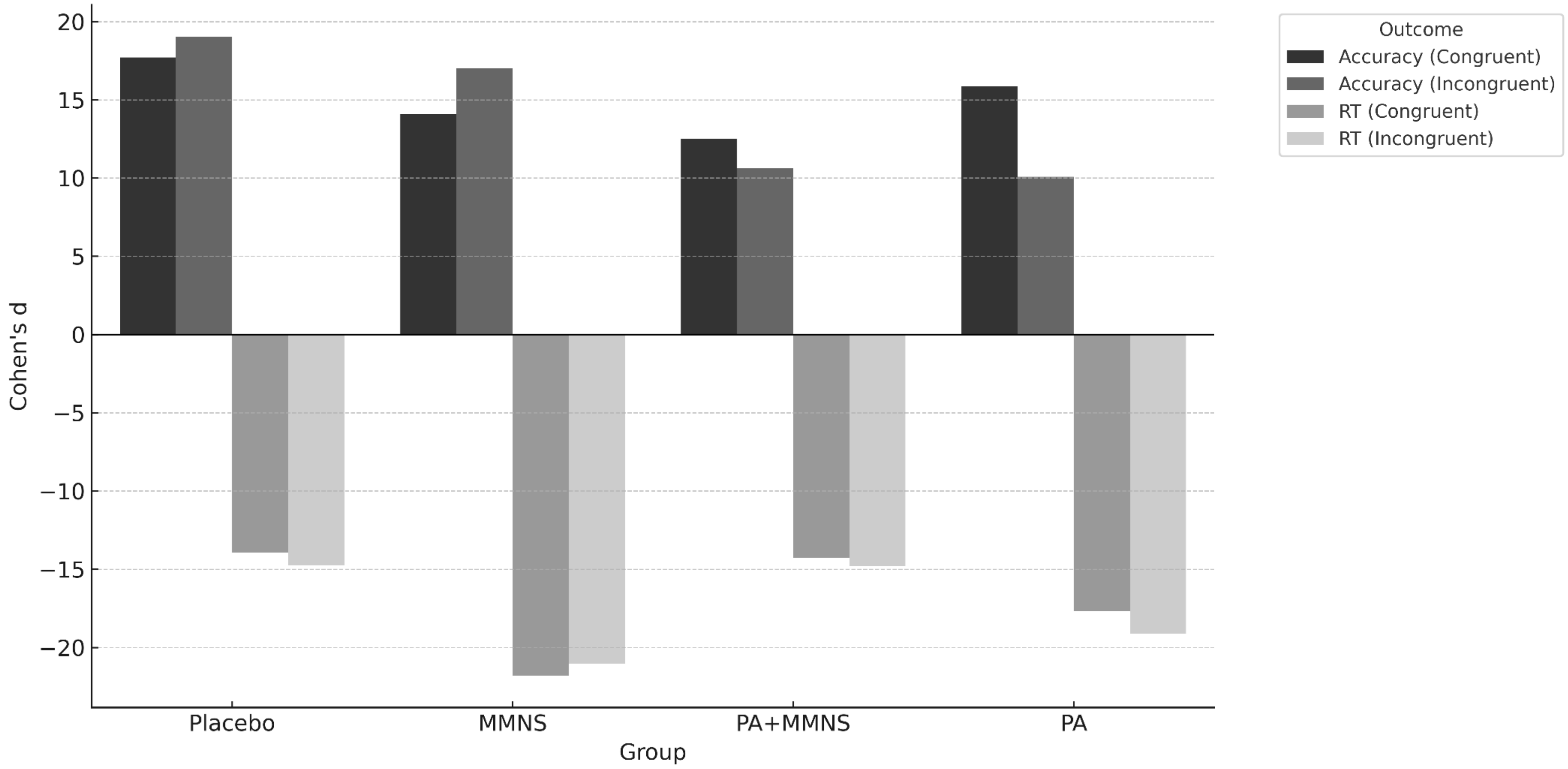

Figure 3: Cohen's d effect sizes for within-group changes from baseline (T1) to post-intervention (T3) across four cognitive outcomes. Each bar represents the standardized effect size for changes in accuracy and reaction time (RT) on congruent and incongruent trials, separated by intervention groups. All interventions showed large effect sizes, particularly for accuracy outcomes.
