# Supplementary material for: Effect of a School-Based Physical Activity and Multi-Micronutrient Supplementation Intervention on Cognitive Function and Academic Achievement Among Schoolchildren in Tanzania: Secondary Outcome from the KaziAfya Cluster-Randomized Controlled Trial
Source: Int J Environ Res Public Health. 2025 Aug 27;22(9):1335. doi: 10.3390/ijerph22091335 (PMC12469510; doi:10.3390/ijerph22091335)
Supplement: Supplementary file 1 [file ijerph-22-01335-s001.zip › ijerph-3702980-supplementary/Table S1_Content of the multi-micronutrient supplementation chewing tablets.pdf]

Table S1: Content of the multi-micronutrient supplementation chewing tablets

No	Nutrient	Average per 1 tablet
1	b-carotene (as BetTab 20%S)	3.6 mg
2	Vitamin D	400 IU / 10 mcg
3	Vitamin E	9 mg TE
4	Vitamin K	30 mcg
5	Vitamin C	60 mg
6	Vitamin B1 Thiamine	1.1 mg
7	Vitamin B2 Riboflavin	1.3 mg
8	Vitamin B6 Pyriodoxine	0.5 mg
9	Vitamin B12	1.2 mcg
10	Folic Acid	200 mcg
11	Niacinamide	8 mg
12	Iron (added as Fe-EDTA)	8 mg
13	Zinc (added as Zinc Oxide)	5 mg
14	Selenium (added as Sodium Selenite Anhydrous	20 mcg
15	Iodine (added as Potassium Iodate)	100 mcg
