# Supplementary material for: Effect of a School-Based Physical Activity and Multi-Micronutrient Supplementation Intervention on Cognitive Function and Academic Achievement Among Schoolchildren in Tanzania: Secondary Outcome from the KaziAfya Cluster-Randomized Controlled Trial
Source: Int J Environ Res Public Health. 2025 Aug 27;22(9):1335. doi: 10.3390/ijerph22091335 (PMC12469510; doi:10.3390/ijerph22091335)
Supplement: Supplementary file 1 [file ijerph-22-01335-s001.zip › ijerph-3702980-supplementary/Table S2_Descriptive characteristics and baseline differences between groups..pdf]

Table S2: Descriptive characteristics and baseline differences between groups.

**Child characteristics**	**Interventions**				
	**Placebo**<br>*M (95% CI)*	**MMNS**<br>*M (95% CI)*	**PA+MMNS**<br>*M (95% CI)*	**PA**<br>*M (95% CI)*	***p*-value**
Female, n (%)	75 (60.00%)	95 (48.47%)	86 (64.66%9	73 (62.93%)	
Male, n (%)	50 (40.00%)	101 (51.53%)	47 (35.34%)	43 (37.07%)	
Stunting	20 (16.00%)	39 (19.90%)	36 (27.07%)	30 (25.86%9	**0.06**
Age (Years)	9.33 (9.20, 9.46)	9.70 (9.57, 9.829	10.4 (10.3, 10.5)	9.77 (9.63, 9.91)	**0.00**
Height (cm)	127.02 (126.00, 128.00)	129.95 (129.00, 131.00)	132.49 (132.00, 133.00)	128.68 (128.00, 130.00)	**0.00**
Weight (kg)	25.60 (25.10, 26.20)	27.5 (27.00, 28.00)	29.5 (29.00, 30.00)	27.5 (27.10, 27.90)	**0.00**
BMI (kg/m^2)	11.1 (9.25,13.0)	14.3 (13.2, 15.5)	9.70 (7.43, 12.0)	9.50 (7.23,11.8)	0.19
zBMI	-0.53 (-0.62, -0.44)	-0.37 (-0.45, -0.29)	-0.23 (-0.31, -0.14)	-0.13 (-.0.21, -0.05)	**0.01**
**Cognitive function**					
Accuracy (congruent stimuli)	0.87 (0.85, 0.88)	0.89 (0.88, 0.91)	0.93 (0.92, 0.94)	0.93 (0.93, 0.94)	**0.00**
Accuracy (incongruent stimuli)	0.81 (0.79, 0.83)	0.85 (0.83, 0.86)	0.91 (0.89, 0.92)	0.83 (0.81, 0.85)	**0.00**
Reaction time (congruent stimuli)	1183.02 (1159.00, 1207.00)	1163.18 (1144.00, 1182.00)	1078.44 (1059.00, 1098.00)	1173.38 (1151.00, 1195.00)	**0.00**
Reaction time (incongruent stimuli)	1239.98 (1213.00, 1267.00)	1216.11 (1195.00, 1237.00)	1137.30 (1116.00, 1159.00)	1228.74 (1206.00, 1251.00)	**0.01**
**Academic achievement**					
End of year results	56.75 (54.60, 58.90)	62.25 (60.30, 64.20)	58.48 (56.90, 60.10)	58.42 (56.80, 60.00)	0.16
Kiswahili Language	63.72 (61.20, 66.30)	68.33 (66.10, 70.60)	60.79 (58.90, 62.70)	68.49 (66.40, 70.60)	**0.05**
Mathematics	49.78 (47.40, 52.20)	56.17 (54.10, 58.20)	56.17 (54.40, 58.00)	48.34 (46.80, 49.90)	**0.00**
