# Supplementary material for: Effect of a School-Based Physical Activity and Multi-Micronutrient Supplementation Intervention on Cognitive Function and Academic Achievement Among Schoolchildren in Tanzania: Secondary Outcome from the KaziAfya Cluster-Randomized Controlled Trial
Source: Int J Environ Res Public Health. 2025 Aug 27;22(9):1335. doi: 10.3390/ijerph22091335 (PMC12469510; doi:10.3390/ijerph22091335)
Supplement: Supplementary file 1 [file ijerph-22-01335-s001.zip › ijerph-3702980-supplementary/Table S3_ICC analysisi.pdf]

Table S3: Intraclass Correlation Coefficients (ICC) for class-level clustering in accuracy outcomes (congruent stimuli). Adjusted ICC accounts for fixed effects in the model. ICCs were estimated using the performance package in R.

**Outcome Variable**	**Adjusted ICC**	**Unadjusted ICC**	**95% CI**	**Interpretation**
Accuracy (Congruent stimuli, T3)	0.029	0.020	Not reported	low class-level clustering
Accuracy (Incongruent stimuli, T3)	NA	NA	-	ICC could not be estimated due to singular model; random effect variance ≈ 0
Reaction time congruent stimulus	0.116	0.097	Not reported	Moderate class-level variance
Reaction time incongruent stimulus	0.111	0.095	Not reported	Moderate class-level variance
Ends of the year results	0.324	0.211	Not reported	Substantial class-level clustering
Kiswahili	0.163	0.094	Not reported	Moderate clustering
Mathematics	0.315	0.239	Not reported	Substantial class-level clustering
