# Supplementary material for: Effect of a School-Based Physical Activity and Multi-Micronutrient Supplementation Intervention on Cognitive Function and Academic Achievement Among Schoolchildren in Tanzania: Secondary Outcome from the KaziAfya Cluster-Randomized Controlled Trial
Source: Int J Environ Res Public Health. 2025 Aug 27;22(9):1335. doi: 10.3390/ijerph22091335 (PMC12469510; doi:10.3390/ijerph22091335)
Supplement: Supplementary file 1 [file ijerph-22-01335-s001.zip › ijerph-3702980-supplementary/Table S4_Intervention and age interaction docx.pdf]

Table S4: Mixed multiple linear regression analyses, adjusted for potential confounders, to explain the effect of the three intervention conditions on cognitive function and academic achievement, in comparison to the placebo control group

**Explanatory variables**	**Mixed multiple linear regression**		
	**Adjusted**		
	**Estimate**	**S.E**	***p-value***
**Accuracy at T3 (congruent stimuli)**			
MMNS	-0.00	0.00	0.50
PA	-0.00	0.00	0.09
PA+MMNS	-0.00	0.04	0.18
Sex (0 = girls, 1 = boys)	-0.00	0.00	**0.00**
Age (years)	-0.00	0.00	**0.02**
zBMI	0.00	0.00	0.73
Stunting (0 = not stunted, 1 = stunted)	-0.01	0.00	0.12
MVPA (0 = not meet, 1 = meet)	0.00	0.00	0.76
Low SES	-0.00	0.00	0.06
Middle SES	-0.00	0.00	0.17
Haemoglobin	0.01	0.00	**0.00**
Dietary diversity (WDDS)	-0.00	0.00	0.42
Food security (HHS)	-0.00	0.00	0.68
Baseline accuracy (congruent stimuli)	0.09	0.00	**0.00**
Intervention*Age-years_exact_T1	0.00	0.00	**0.17**
**Accuracy at T3 (incongruent stimuli)**			
MMNS	0.00	0.01	0.85
PA	-0.02	0.01	0.12
PA+MMNS	-0.01	0.01	**0.00**
Sex	-0.01	0.00	0.24
Age (years)	-0.01	0.00	**0.02**
zBMI	-0.00	0.04	0.40
Stunting (0 = not stunted, 1 = stunted)	-0.00	0.01	0.47
MVPA (0 = not meet, 1 = meet)	-0.00	0.01	0.80
Low SES	-0.01	0.01	0.10
Middle SES	-0.01	0.01	0.05
Haemoglobin	0.00	0.01	0.55
Dietary diversity (WDDS)	-0.00	0.00	0.13
Food security (HHS)	-0.00	0.01	0.97
Baseline accuracy (incongruent stimuli)	0.20	0.02	**0.00**
Intervention*Age-years_exact_T1	0.02	0.00	**0.00**
**Reaction time at T3 (congruent stimuli)**			
MMNS	-14.50	31.76	0.65
PA	-23.98	30.78	0.43
PA+MMNS	-32.14	187.23	0.86
Sex	-106.97	21.11	**0.00**
Age (years)	-10.28	10.29	0.32
zBMI	0.89	9.52	0.92
Stunting (0 = not stunted, 1 = stunted)	14.57	23.91	0.54
MVPA (0 = not meet, 1 = meet)	31.61	37.17	0.39

Low SES	10.74	23.91	0.65
Middle SES	-4.79	23.55	0.84
Haemoglobin	-2.16	8.98	0.81
Dietary diversity (WDDS)	-10.47	11.79	0.37
Food security (HHS)	-13.26	12.82	0.30
Baseline reaction time (congruent stimuli)	0.23	0.04	**0.00**
Intervention*Age-years_exact_T1	4.85	17.90	0.78
**Reaction time at T3 (incongruent stimuli)**			
MMNS	-7.30	31.87	0.82
PA	-22.94	30.74	0.45
PA+MMNS	8.03	187.16	0.96
Sex	-114.88	20.91	**0.00**
Age (years)	-5.35	10.43	0.61
zBMI	-0.38	9.60	0.96
Stunting (0 = not stunted, 1 = stunted)	19.04	23.83	0.42
MVPA (0 = not meet, 1 = meet)	25.08	37.10	0.50
Low SES	7.84	23.91	0.74
Middle SES	-7.13	23.55	0.76
Haemoglobin	1.39	8.95	0.87
Dietary diversity (WDDS)	-10.04	11.77	0.39
Food security (HHS)	-11.81	12.82	0.36
Baseline reaction time (incongruent stimuli)	0.22	0.03	**0.00**
Intervention*Age-years_exact_T1	1.01	17.87	0.95
**End-of-the-year results at T3**			
MMNS	8.61	10.28	0.40
PA	-45.02	9.85	**0.00**
PA+MMNS	-4.30	58.51	0.94
Sex	11.80	6.56	0.07
Age (years)	-5.21	3.42	0.13
zBMI	0.94	3.05	0.76
Stunting (0 = not stunted, 1 = stunted)	8.37	7.63	0.27
MVPA (0 = not meet, 1 = meet)	10.03	11.38	0.37
Low SES	-3.84	7.50	0.60
Middle SES	-8.29	7.32	0.25
Haemoglobin	-2.46	2.84	0.38
Dietary diversity (WDDS)	-0.09	3.86	0.98
Food security (HHS)	1.14	4.03	0.77
Baseline end-of-the-year results	2.05	0.14	**0.00**
Intervention*Age-years_exact_T1	2.35	5.57	0.67
**Performance in Kiswahili at T3**			
MMNS	12.17	2.10	**0.00**
PA	-3.86	2.02	0.05
PA+MMNS	-3.22	11.95	0.78
Sex	0.59	1.35	0.65
Age (years)	-0.96	0.68	0.15
zBMI	0.57	0.63	0.36
Stunting (0 = not stunted, 1 = stunted)	0.09	1.57	0.94

MVPA (0 = not meet, 1 = meet)	0.71	2.33	0.76
Low SES	0.40	1.55	0.80
Middle SES	-2.28	1.50	0.12
Haemoglobin	-1.04	0.58	0.07
Dietary diversity (WDDS)	-0.22	0.79	0.77
Food security (HHS)	-0.16	0.83	0.84
Baseline performance in Kiswahili	0.35	0.02	**0.00**
Intervention*Age-years_exact_T1	0.99	1.13	0.38
**Performance in mathematics at T3**			
MMNS	-3.06	2.33	0.19
PA	0.99	2.09	0.64
PA+MMNS	-28.39	12.78	**0.02**
Sex	2.29	1.34	0.08
Age (years)	-2.00	0.68	**0.00**
zBMI	-0.28	0.63	0.64
Stunting (0 = not stunted, 1 = stunted)	1.03	1.56	0.51
MVPA (0 = not meet, 1 = meet)	1.50	2.37	0.52
Low SES	-0.34	1.59	0.82
Middle SES	-0.49	1.54	0.75
Haemoglobin	-0.02	0.62	0.97
Dietary diversity (WDDS)	0.53	0.81	0.51
Food security (HHS)	-0.17	0.83	0.82
Baseline performance in mathematics	0.29	0.02	**0.00**
Intervention*Age-years_exact_T1	2.76	1.19	**0.02**
