# Supplementary material for: Effect of a School-Based Physical Activity and Multi-Micronutrient Supplementation Intervention on Cognitive Function and Academic Achievement Among Schoolchildren in Tanzania: Secondary Outcome from the KaziAfya Cluster-Randomized Controlled Trial
Source: Int J Environ Res Public Health. 2025 Aug 27;22(9):1335. doi: 10.3390/ijerph22091335 (PMC12469510; doi:10.3390/ijerph22091335)
Supplement: Supplementary file 1 [file ijerph-22-01335-s001.zip › ijerph-3702980-supplementary/Table S5_IVF.analysis.pdf]

Table S5: Variance Inflation Factors (VIFs) for Covariates

Variables	GVIF	DF	GVIF^(1/(2*Df))
**Accuracy at T3 (congruent stimuli)**			
MMNS	1.96	1	1.400
PA	1.64	1	1.28
PA+MMNS	1.92	1	1.38
Age (Years)	1.11	1	**1.05**
Sex	1.11	1	**1.05**
MVPA	1.05	1	1.02
Stunting	1.07	1	1.03
SES (3 levels)	1.08	2	1.02
zBMI	1.03	1	1.01
Food insecurity (HHS)	1.04	1	1.02
Haemoglobin	1.01	1	1.00
Dietary diversity (WDDS)	1.07	1	1.03
Baseline accuracy (congruent stimuli)	1.04	1	1.02
**Accuracy at T3 (incongruent stimuli)**			
MMNS	1.79	1	1.33
PA	1.72	1	1.31
PA+MMNS	1.78	1	1.33
Sex	1.07	1	1.03
Age (Years)	1.19	1	1.09
MVPA	1.05	1	1.02
Stunting	1.05	1	1.02
SES (3 levels)	1.08	1	1.02
zBMI	1.03	1	1.01
Food insecurity (HHS)	1.03	1	1.01
Haemoglobin	1.04	1	1.02
Dietary diversity (WDDS)	1.07	1	1.03
Baseline accuracy (incongruent stimuli)	1.09	1	1.04
**Reaction time at T3 (congruent stimuli)**			
MMNS	2.05	1	1.43
PA	1.59	1	1.26
PA+MMNS	2.03	1	1.42
Sex	1.17	1	1.08
Age (Years)	1.15	1	1.07
MVPA	1.05	1	1.02
Stunting	1.08	1	1.04
SES (3 levels)	1.09	2	1.02
zBMI	1.04	1	1.02
Food insecurity (HHS)	1.04	1	1.02
Haemoglobin	1.02	1	1.01
Dietary diversity (WDDS)	1.07	1	1.03
Baseline reaction time (congruent stimuli)	1.09	1	1.04
**Reaction time at T3 (incongruent stimuli)**			
MMNS	2.05	1	1.43
PA	1.59	1	1.26
PA+MMNS	2.01	1	1.41

Sex	1.14	1	1.07
Age (Years)	1.17	1	1.08
MVPA	1.05	1	1.02
Stunting	1.08	1	1.04
SES (3 levels)	1.09	2	1.02
zBMI	1.04	1	1.02
Food insecurity (HHS)	1.05	1	1.02
Haemoglobin	1.01	1	1.00
Dietary diversity (WDDS)	1.07	1	1.03
Baseline reaction time (incongruent stimuli)	1.08	1	1.04
**End-of-the-year results at T3**			
MMNS	2.15	1	1.46
PA	1.52	1	1.23
PA+MMNS	2.11	1	1.45
Sex	1.14	1	1.06
Age (Years)	1.16	1	1.08
MVPA	1.05	1	1.02
Stunting	1.08	1	1.04
SES (3 levels)	1.07	2	1.01
zBMI	1.05	1	1.02
Food insecurity (HHS)	1.05	1	1.02
Haemoglobin	1.01	1	1.00
Dietary diversity (WDDS)	1.09	1	1.04
Baseline end-of-the-year results	1.11	1	1.05
**Performance in Kiswahili at T3**			
MMNS	2.13	1	1.46
PA	1.53	1	1.23
PA+MMNS	2.06	1	1.44
Sex	1.13	1	1.06
Age (Years)	1.15	1	1.07
MVPA	1.05	1	1.02
Stunting	1.08	1	1.04
SES (3 levels)	1.07	2	1.01
zBMI	1.06	1	1.03
Food insecurity (HHS)	1.05	1	1.02
Haemoglobin	1.02	1	1.01
Dietary diversity (WDDS)	1.10	1	1.05
Baseline performance in Kiswahili	1.09	1	1.05
**Performance in mathematics at T3**			
MMNS	2.76	1	1.66
PA	1.64	1	1.28
PA+MMNS	2.64	1	1.62
Sex	1,11	1	1.05
Age (Years)	1,13	1	1.06
MVPA	1.06	1	1.03
Stunting	1.09	1	1.04
SES (3 levels)	1.12	2	1.02

zBMI	1.04	1	1.02
Food insecurity (HHS)	1.07	1	1.03
Haemoglobin	1.03	1	1.01
Dietary diversity (WDDS)	1.12	1	1.06
Baseline performance in mathematics	1.11	1	1.05
