# Supplementary material for: Effect of a School-Based Physical Activity and Multi-Micronutrient Supplementation Intervention on Cognitive Function and Academic Achievement Among Schoolchildren in Tanzania: Secondary Outcome from the KaziAfya Cluster-Randomized Controlled Trial
Source: Int J Environ Res Public Health. 2025 Aug 27;22(9):1335. doi: 10.3390/ijerph22091335 (PMC12469510; doi:10.3390/ijerph22091335)
Supplement: Supplementary file 1 [file ijerph-22-01335-s001.zip › ijerph-3702980-supplementary/Table S7_ Pairwise comparisons of covariates at time point 3 between intervention groups.pdf]

Table S7: Pairwise comparisons of covariates at time point 3 between intervention groups

**Comparison**	**Adjusted p-value**
**Age**	
Placebo vs MMNS	0.189
Placebo vs PA+MMNS	**0.000**
Placebo vs PA	0.094
MMNS vs PA+MMNS	**0.003**
MMNS vs PA	0.931
PA+MMNS vs PA	**0.013**
**BMI**	
Placebo vs MMNS	0.624
Placebo vs PA+MMNS	**0.018**
Placebo vs PA	0.176
MMNS vs PA+MMNS	0.179
MMNS vs PA	0.708
PA+MMNS vs PA	0.844
**Accuracy (incongruent stimuli)**	
Placebo vs MMNS	0.983
Placebo vs PA+MMNS	0.507
Placebo vs PA	0.114
MMNS vs PA+MMNS	0.649
MMNS vs PA	**0.026**
PA+MMNS vs PA	**0.001**
**Reaction time (congruent stimuli)**	
Placebo vs MMNS	0.026
Placebo vs PA+MMNS	0.037
Placebo vs PA	0.411
MMNS vs PA+MMNS	0.999
MMNS vs PA	0.729
PA+MMNS vs PA	0.714
**End of the year results**	
Placebo vs MMNS	**0.007**
Placebo vs PA+MMNS	0.172
Placebo vs PA	**0.000**
MMNS vs PA+MMNS	0.746
MMNS vs PA	0.728
PA+MMNS vs PA	0.258
**Kiswahili**	
Placebo vs MMNS	**0.000**
Placebo vs PA+MMNS	0.998
Placebo vs PA	0.941
MMNS vs PA+MMNS	**0.000**
MMNS vs PA	**0.000**
PA+MMNS vs PA	0.977
**Mathematics**	
Placebo vs MMNS	**0.000**
Placebo vs PA+MMNS	**0.000**
Placebo vs PA	0.156

MMNS vs PA+MMNS	0.809
MMNS vs PA	0.426
PA+MMNS vs PA	0.152
