# Supplementary material for: Effect of a School-Based Physical Activity and Multi-Micronutrient Supplementation Intervention on Cognitive Function and Academic Achievement Among Schoolchildren in Tanzania: Secondary Outcome from the KaziAfya Cluster-Randomized Controlled Trial
Source: Int J Environ Res Public Health. 2025 Aug 27;22(9):1335. doi: 10.3390/ijerph22091335 (PMC12469510; doi:10.3390/ijerph22091335)
Supplement: Supplementary file 1 [file ijerph-22-01335-s001.zip › ijerph-3702980-supplementary/Table S8_Unadjusted mixed multiple linear regression analyse.pdf]

Table S8. Unadjusted mixed multiple linear regression analyses of the effect of the three intervention conditions on cognitive function and academic achievement, in comparison to the placebo control

Explanatory variables	Mixed multiple linear regression		
	Unadjusted		
	**Estimate**	**SE**	***p-value***
**Accuracy (congruent stimuli)**			
MMNS	-0.00	0.00	0.92
PA + placebo	-0.00	0.00	0.25
PA+MMNS	0.00	0.00	0.52
**Accuracy (incongruent stimuli)**			
MMNS	0.01	0.01	0.38
PA + placebo	-0.03	0.01	**0.03**
PA+MMNS	0.02	0.01	0.13
**Reaction time (congruent stimuli)**			
MMNS	-54.47	31.05	0.07
PA + placebo	-23.86	30.15	0.43
PA+MMNS	-9.15	32.89	0.78
**Reaction time (incongruent stimuli)**			
MMNS	-58.62	33.02	0.07
PA + placebo	-24.72	32.08	0.44
PA+MMNS	-5.342	35.02	0.87
**End-of-the-year academic results**			
MMNS	27.52	11.28	**0.01**
PA + placebo	-19.00	10.97	0.08
PA+MMNS	29.48	11.88	**0.01**
**Performance in Kiswahili**			
MMNS	15.78	2.29	**<0.01**
PA + placebo	1.69	2.22	0.44
PA+MMNS	7.35	2.41	**<0.01**
**Performance in mathematics**			
MMNS	1.09	2.44	0.65
PA + placebo	4.06	2.13	**0.05**
PA+MMNS	5.04	2.92	0.08

Notes. PA = physical activity, MMNS = multi-micronutrient supplementation, SE = standard error. All estimates are from linear mixed models, including treatment groups (intervention and placebo) as fixed effects, and school classes as random effects.
